# *In Silico* Identification of ANKRD22 as a Theragnostic Target for Pancreatic Cancer and Fostamatinib's Therapeutic Potential

**DOI:** 10.7150/ijms.105193

**Published:** 2025-03-19

**Authors:** Huong Thi Luu Kim Huynh, Hendrick Gao-Min Lim, Yuan-Chii Gladys Lee, Thien-Vy Phan, Thanh-Hoa Vo, Chien-Hsin Chen, Alexander T.H. Wu

**Affiliations:** 1 International PhD Program for Translational Science, College of Medical Science and Technology, Taipei Medical University, Taipei 11031, Taiwan.; 2 Graduate Institute of Biomedical Informatics, College of Medical Science and Technology, Taipei Medical University, Taipei 11031, Taiwan.; 3 Department of Medical Research, Tzu Chi Hospital Indonesia, Pantai Indah Kapuk, Greater Jakarta, Indonesia 14470.; 4 Department of Pharmacy, Nguyen Tat Thanh University, Ho Chi Minh City 700000, Vietnam.; 5 University of Health Sciences, Vietnam National University Ho Chi Minh City, Ho Chi Minh City 700000, Vietnam.; 6 Department of Surgery, College of Medicine, Taipei Medical University, Taipei 11031, Taiwan.; 7 Department of Colorectal Surgery, Wan Fang Hospital, Taipei Medical University, Taipei 11031, Taiwan.; 8 TMU Research Center of Cancer Translational Medicine, Taipei Medical University, Taipei 11031, Taiwan.; 9 The PhD Program of Translational Medicine, College of Medical Science and Technology, Taipei Medical University, Taipei 11031, Taiwan.; 10 Graduate Institute of Medical Sciences, National Defense Medical Center, Taipei 114, Taiwan.; 11 Taipei Heart Institute (THI), Taipei Medical University, Taipei, Taiwan.

**Keywords:** bioinformatics, pancreatic cancer (PC), KRAS, ANKRD22, fostamatinib

## Abstract

Pancreatic cancer (PC) is one of the most tremendously malignant cancers with a poor prognosis, especially when it advances to metastasis. Besides, PC patients have encountered resistance to recent therapeutic approaches. In recent work, we effectively determined ANKRD22 by re-analyzing RNA-seq datasets from cell lines and human tissues deriving from PC. We demonstrated that ANKRD22 expression was remarkably high in the PC group compared to the normal group at both gene expression and protein levels. ANKRD22 resulted in a worse overall survival (OS) rate of PC patients (HR = 1.7, p = 0.0082). Intriguingly, ANKRD22 was statistically highly expressed in the mutated KRAS group relative to the wildtype group (p < 0.05). Similarly, compared to the wildtype TP53, in the mutated TP53, ANKRD22 also significantly expressed (p < 0.05); their concurrent expression, ANKRD22 and KRAS; ANKRD22 and TP53 exacerbated the survival outcome relative to the co-expression of low ANKRD22 and unaltered genes (p < 0.001; HR > 2.6). We explored the potential pathways and biological processes ANKRD22 might not only contribute to promoting PC, including cell-cycle regulation, E2F1 targets, and apoptosis but also foster the dissemination of PC by involve in invasion and migration processes. In the investigation of drugs that might target ANKRD22, we figured out fostamatinib. Molecular docking and molecular dynamic simulation (MDs) techniques provided extensive insights into the binding mode of ANKRD22 and fostamatinib. ANKRD22 exhibited strong binding affinity (ΔG = -7.0 kcal/mol in molecular docking and ∆G_bind_ = -38.66 ± 6.09 kcal/mol in MDs). Taken together, ANKRD22 could be a promising theragnostic target that might be inhibited by fostamatinib, thereby suppressing PC growth.

## Introduction

The 5-year survival rate of PC patients in the US is 10% 1, reflecting that PC is one of the most lethal and aggressive cancers. Various factors leading to this dismal rate include asymptomatic or non-specific manifestations, lack of markers or diagnostic methods for early detection, drug resistance, and less response to the current standard treatment 2, 3. Metastasis refers to the primary tumor migrating from its original site to evade and localize at the foreign organs 4, 5. This process is also considered to contribute to the malignancy of PC. Indeed, patients diagnosed at metastatic stages have a worse 5-year survival rate of only 3% 1. PC progresses from pancreatic intraepithelial neoplasia (PanIN) to metastasis, and pancreatic adenocarcinoma (PAAD) is the most prevalent and deadliest tumor type itself, accounting for 85% of all cases 3, 6. It has been reported that KRAS is the PC-driven gene; its mutation contributes to > 90% of PC patients. Despite the numerous efforts to explore the underlying mechanism of PC coupled with attempts to investigate the novel therapeutic approaches that have been conducted, PC is still a refractory disease 7, 8. Consequently, there is an urgent need to explore a new theragnostic molecule that can benefit the diagnosis and target therapy for PC, especially for metastatic PC, which exhibits the lowest survival rate.

Ankyrin repeat domain 22, ANKRD22, has a unique structure encompassing four identical L-shaped ankyrin motifs, allowing it to interact with a wide range of molecules. Therefore, its relevant biological pathways and functions can be involved in various diseases, including cancer 9-11. ANKRD22 plays a dual role in cancer development as a cancer suppressor or tumor supporter. For instance, in colorectal cancer, ANKRD22 extensively contributed to metabolic reprogramming, leading to colorectal cancer growth 9. Similarly, ANKRD22 facilitated the proliferation of non-small lung cancer cells, and its function might be via the upregulation of E2F1 transcription factor, which is well related to the cell cycle process 10. In contrast, regarding prostate cancer, ANKRD22 is lowly expressed in the tumor group relative to the normal group, and the unfavorable correlation between ANKRD22 and the survival outcome of the patients was identified. This study revealed the anti-tumorigenesis role of ANKRD22 11. Interestingly, ANKRD22 was recognized as a promising marker that might help in the early diagnosis of PC 12. ANKRD22 has been demonstrated to have a significantly high expression in KRAS-addicted cell lines; knockdown ANKRD22 diminished E-cad expression and elevated Caspade-3, one of the markers for apoptosis. These findings highlighted the correlation between ANKRD22 and aberrant KRAS and indicated the possibility that ANKRD22 relates to the epithelial-mesenchymal transition process 13. Unfortunately, the mechanism ANKRD22 might exploit in PC progression and metastasis remains elusive. As a result, further investigation is needed to explore its potential contribution to PC.

Leveraging a well-established drug whose function and safety were already known and then exploring its extra function to apply that efficacy to other targets would save time and be cost-effective. Those are the purposes of drug repurposing 14. Recent evidence has shown that fostamatinib emerged as a promising candidate for drug repositioning, particularly in anti-cancer therapeutics. Fostamatinib is initially prescribed for immune thrombocytopenia patients to inhibit spleen tyrosine kinase (SYK) 15. In hepatocellular carcinoma (HCC), it served as an inhibitor of tumor growth *in vitro* and *in vivo*. Fostamatinib might regulate the JAK/STAT, PI3K/AKT, and MAPK/ERK signaling pathways 16. As observed in glioma cancer, the synergistic impact between fostamatinib and temozolomide further inhibited the viability of glioma stem cells 17. These results unveiled another function of fostamatinib apart from its intended target.

In this study, we aimed to elucidate the oncogenic function of ANKRD22 in PC, provide insights into the progression and metastasis of PC to which ANKRD22 might contribute, and repurpose fostamatinib for treating PC.

## Materials and methods

### Acquisition of RNA expression dataset

The publicly available PC datasets, GSE149103 and GSE63124, were downloaded from the GEO database 18. The GSE149103 dataset consisted of RNA-sequence data of three distinct properties of human PC cell lines: human pancreas normal epithelial cells (HPNE), PANC-1 derived from the head of the pancreas as primary PC cells, and CAPAN-1, the metastatic PC cells (mPC) deriving from liver metastasis. In contrast, the GSE63124 dataset included RNA-sequence data from two mPC patients, patient A13 with lung metastasis and patient A38 with liver, lung, and peritoneal metastasis 19, 20. These organs are the most common destinations where PC cells migrate and reside. Hence, analyzing this dataset may identify an aggressive gene as a critical player in mPC 2, 3.

### Identification of differentially expressed genes (DEGs)

Identifying DEGs between groups of two datasets was conducted using the Galaxy platform 21. Firstly, we evaluated the quality control of the raw data by running the FastQC tool vers. 0.12.1, followed by the Trimmomatic tool vers. 036 to remove any adapters and reads with an average quality score of less than 20 22, 23. Next, Hisat2 version 2.2.1 (Hierarchical Indexing for Spliced Alignment of Transcripts) was used to map the reads with the human reference genome (Gencode, release 38, hg38) with the default parameters 24. Raw counts were then obtained with Featurecounts version 2.0.3 25. Finally, DEGs were identified between non-metastatic (HPNE and PANC-1) and metastatic (CAPAN-1) cell lines for the GSE149103 dataset and between each pairwise of metastatic sites for the GSE63124 dataset (Lung-Liver, Peritoneal-Liver, and Peritoneal-Lung) by using Limma version 3.48.0, in which the output file of annotateMyIDs vers. 3.16.0 was utilized to annotate each gene 26, 27. The count per million CPM value was set at 0.046 and 0.007 for GSE149103 and GSE63124, respectively, to filter the lowly expressed genes. Besides, |log2 fold change| > 1 and adjusted (adj.) p-value of < 0.01 were also applied for filtering DEGs. DEGs with an FC value > 2 were considered upregulated genes, and DEGs with an FC value < 2 were considered downregulated.

Ultimately, the Bioinformatics and Evolutionary Genomics web tool created Venn diagrams between two datasets to identify overlapped DEGs.

### Analysis of the association of the most significant upregulated overlapped genes in the PAAD cohort

GEPIA (Gene Expression Profiling Interactive Analysis version 2, a valuable online web tool using transcriptomic data from The Cancer Genome Atlas (TCGA) and Genotype-Tissue Expression projects (GTEx), was employed to investigate the correlation between the expression of overlapping upregulated genes and the overall survival (OS) rate as well as their expressions in different tumor stages in PAAD cohort 28. The Mantel-Cox test was used to examine the OS rate with a median cutoff chosen to split the PAAD cohort into high and low gene expression groups for each overlapping upregulated gene. Hazard ratio (HR) > 1 and p-value of < 0.05 were considered significantly riskier for patients with high gene expression than those with low gene expression. The one-way ANOVA test was used to analyze gene expression across cancer stages. Furthermore, to examine ANKRD22 expression in the PAAD group relative to the normal group, the box-plot module was used with |Log2FC| cutoff = 1, and the p-value cutoff = 0.01 was performed.

### Investigation of ANKRD22 expression at the protein level in PAAD patients

The human protein Atlas (HPA) was used to examine ANKRD22 protein expression in PAAD patients. Immunohistochemical (IHC) results showed protein expressions classified into four categories based on the fraction of stained cells (less than 25%, 25%-75%, and greater than 75%) and staining intensity (negative, weak, moderate, and strong). The categories were as follows: "not-detected" category included negative or weak and less than 25%; "low" had weak and either 25%-75% or greater than 75%, moderate and less than 25%; "medium" included moderate and either 25%-75% or greater than 75%, strong and less than 25%; and "high" included strong and either 25%-75% or greater than 75% 29

### Discovering potential pathways or biological processes that ANKRD22 might involve in mPC initiation

To examine the potential role of ANKRD22 in the development of mPC, we performed Gene Set Enrichment Analysis (GSEA) and Ingenuity Pathway Analysis (IPA). These two software tools are based on existing knowledge or pre-defined gene sets and provide potential biological pathways and processes 30, 31. The input files contain two groups, high and low ANKRD22 expression groups. These groups were obtained from the TCGA database 32.

We used Oncogenic Signature, Hallmark, and KEGG gene sets for GSEA analysis. The parameters chosen were Number of permutations 1000, collapse to gene symbols, the chip platform "Human_Ensemble_Gene_ID_MSigDB.v2023.2.Hs.chip", and Permutation type was "gene_set". The significantly enriched hallmarks and signature pathways were identified based on a normalized enrichment score (NES) > 1, the p-value < 0.05, and FDR (false discovery rate) < 5%. Finally, we used ImageGP visualization tools to illustrate the pathways 33.

DEGs between high and low ANKRD22 expression groups were uploaded to IPA for analysis. DEGs met the cutoff of |Log2FC|> 1, and the p-value < 0.05 were further analyzed in this software.

### Investigation of potential drugs that might interact with ANKRD22

To explore drugs that might target ANKRD22, we accessed two databases, Drugbank and Chembl. These two valuable databases provide comprehensive information on drugs and their targets 34, 35.

### Investigation of the interaction between ANKRD22 and Fostamatinib

To further investigate the interaction between fostamatinib and ANKRD22, we utilized STITCH (Search Tool for Interacting Chemicals, version 5.0). This helpful online tool depicts the potential interactions of over four hundred thousand chemicals with target proteins 36. ANKRD22 and fostamatinib were the input items inserted into STITCH; the medium confidence of 0.4 and no more than 10 interactions for 1st shell and 2nd shell were set to get the protein-protein or protein-chemical interactions.

### Exploring correlations of ANKRD22 and SYK, RIPK4 in the PAAD cohort

TIMER 2.0 is an online tool exploring the correlation between genes, especially target genes and mutated genes, which might play a crucial role in tumor progression 37. Using TIMER 2.0, we aimed to determine the association between ANKRD22 and SYK, RIPK4. The purity-adjusted partial Spearman's rho value with a statistical significance threshold was set as a p-value of < 0.05.

### Identification of ANKRD22 expression in the wildtype and mutated KRAS, TP53 groups

In order to explore the expression of ANKRD22 in the presence of mutated KRAS, TP53 was compared to wildtype groups, TIMER 2.0 was also used. The statistical significance computed by the Wilcoxon test was identified as a p-value of < 0.05.

The cBioPortal for Cancer Genomics is a multi-function web-based tool for performing genetic alteration analyses 38. We utilized this cBioPortal to address information about the proportion of mutated KRAS and TP53 and their impact on the OS rate in the PAAD cohort. The statistical method used for this analysis was the log_rank test; the HRs and 95% clearance interval (95% CI) were identified. This PAAD cohort was derived from the TCGA database with 179 PC patients.

To broaden the association between ANKRD22 co-expressing those altered genes and OS rate in the PAAD cohort, we performed Kaplan-Mier analysis with log_rank test by using GraphPad Prism version 9.5.0 for Windows, GraphPad Software, San Diego, California, USA. Results were regarded statistically significant when they met p < 0.05. The input data was downloaded from TCGA.

### *In silico* molecular docking analysis of fostamatinib, AV023 bound to ANKRD22

Molecular docking is a commonly used tool to investigate binding poses in the interaction between ligands and receptors. The key functions of this method are to determine the best pose and calculate the affinity 39, 40. This substantial method has been seen as an indispensable part of drug repurposing.

In order to examine the potential interactions between fostamatinib and ANKRD22, first, the three-dimensional (3D) predicted structure of ANKRD22 (PDB format) was obtained from AlphaFold 41. Besides, due to the lack of crystal structure of ANKRD22, we employed Cavityplus 2022 to determine the potential binding pocket 42. The cavities were ranked based on druggability and drug scores (the score for druggability assessment). The degree of druggability was classified as strong, with a DurgScore of ≥ 600; medium, with 600 > DrugScore ≥ -180; and weak, with a DrugScore of < -180 43. We then prepared the ANKRD22 protein by converting the PDB format to PDBQT format; after that, deleting H_2_O molecules, adding Kollman charges, and adjusting polar hydrogens were conducted on the Autodock tool vers. 1.5.7.

Noteworthy, a recent study has also conducted molecular docking of AV023 compound binding ANKRD22 to explore its function in gastric mucosal injury 44. Therefore, to further understand the interaction between fostamatinib and ANKRD22, we compared this binding to the binding between AV023 and ANKRD22. The 3D structure of fostamatinib (CID 11671467) in SDF format and the SMILE format of AV023's two-dimensional (2D) structure were downloaded from PubChem 45. This SMILE file was converted to 3D structure (SDF format) using an online SMILES translator and structure file generator. Next, these SDF format files were converted into PDB format using Pymol 46, and then Autodock transformed these PDB files to PDBQT format.

Finally, the interactions of ANKRD22, fostamatinib, and AV023 were investigated using Autodock, and the resultant specific residue lists formed predicted binding pockets generated from Cativity were used. In addition, spacing was set to 1 Å, and the numbers of points in the x, y, and z dimensions were 40 × 40 × 40 Å, respectively. Pymol illustrated the 3D docked ligand-receptor complexes, and the 2D was visualized and analyzed using BIOVIA Discovery Studio 47.

### Molecular dynamics simulations analysis of fostamatinib, AV023 bound to ANKRD22

Molecular dynamics simulation (MDs) is a technique to visualize molecules' movement at the atomic level over time. It can cooperate with molecular docking to provide a comprehensive interaction between ligands and proteins. MDs can avoid atomic perturbation and give more accurate results than molecular docking 48, 49.

MDs was conducted on GROMACS tool version 2021.4 for 150 ns 50. As for ANKRD22 protein preparation, the topology of ANKRD22 was explored using forcefield CHARMM-27 51. The best docking score conformation of ligands was exported in MOL2 format. SwissParam performed the topology of ligands using forcefield CHARMM-36 52. Finally, the topology file of complex protein-ligand was created.

A dodecahedron simulation box was built with a radius of 10 Å from the complex. The system was filled with water (TIP3P model) and equilibrium electrical by adding Na+ or Cl- (concentration NaCl final was 0.15 M). The system then was energy minimized by using the steepest descent with the maximum force of 10 kJ/mol in 100 ps. The system performed NVT (constant particle number, volume, and temperature) equilibration at 100 ps, stabilizing a molecular system at a constant number of particles, volume, and temperature (300 K) 53. Subsequently, the Parrinello-Rahman barostat algorithm ran NPT (constant particle number, pressure, and temperature ) equilibration at 100 ps to stabilize the system's density at the constant temperature and pressure (1 bar) 54. The Verlet algorithm performed MD simulation at 300 K and 1 bar. To restrict hydrogen bonds, the LINCS algorithm was used 55. The other interactions were obtained at a cut-off of 12 Å, and electrostatic interactions were calculated using the Mesh Ewald method. The trajectories of MD simulations were saved every 0.01 ns.

Eventually, RMSD (Root Mean Square Deviation), RMSF (Root Mean Square Fluctuation), R_g_ (Radius of Gyration), and SASA (Solvent Accessible Surface Area) values were calculated based on trajectories of MD simulations by gmx_rms, gmx_rmsf, gmx_gyrate, and gmx_sasa commands of GROMACS to evaluate the stability of complexes. The RMSD carbon backbone of protein (RMSD C_backbone_) and RMSD heavy atoms of ligand (RMSD_nonH_) values present the protein's and ligand's stability or flexibility when bound together 56. The RMSF carbon alpha (RMSF C_α_) determines the balance of residues. SASA is a value to evaluate protein folding and stability 57. The value of R_g_ is an index of protein density representing the protein's compactness 58.

The ∆G_bind_ value, binding free energy between a ligand and a protein, was calculated using the MM/GBSA (Molecular mechanics (MM) with generalized Born and surface area solvation (GBSA)) by gmx_MMPBSA command. The formula for the calculation of ∆G_bind_ is: ∆G_bind_ = ∆E_MM_ + ∆G_sol_ - T∆S = (∆E_int_ + ∆E_vdW_ + ∆E_ele_) + ∆G_sol_ - T∆S, in which E_int_, ∆E_vdW_, and ∆E_ele_ are internal energies, Van der Waals energies, and electrostatic energies, respectively. ∆G_sol_ includes polar and non-polar solvation energy, while T∆S is conformational entropy; thus, the ∆G_bind_ value can be decomposed into relevant interactions to identify key residues. The constant solute dielectric, temperature, and NaCl concentrations were set at 1.0, 298 K, and 0.15 M, respectively 59.

The frequency of hydrogen bonds (H-bond) was analyzed from the trajectories of MD simulation using VMD 1.9.4a55 Open GL216 software 60. A hydrogen bond was defined according to geometric criteria: the hydrogen donor (D) - acceptor (A) distance < 3.5 Å, and the angle of hydrogen D - A > 120^0^ 56.

Principal component analysis (PCA) was investigated to present the atomic motions of apoprotein and protein-ligand complex states 61. PCA was calculated using the g_covar and g_anaeig commands of GROMACS 51. The C_backbone_ moments of protein were represented by eigenvectors (EVs) from the variance matrix data 62. Projecting the two vectors EV1 and EV2 on a diagram showed information about the spatial motion of protein. The porcupine plot was drawn from EV1 data by PyMOL to visualize the direction and magnitude of movements 63.

A free energy landscape (FEL) analysis was also performed to characterize the protein's stable state. The FEL plots were performed and analyzed based on the first two EVs of the apo form of ANKD22 and its complexes with AV023 and fostamatinib 64. R packages and GROMACS built-in and standalone tools were used for all post-MD simulation data analyses.

## Results

### Identification of DEGs between metastatic and non-metastatic cell lines and across metastatic sites of pancreatic cancer

The gene expression data from RNA-seq of the GSE149103 dataset was first analyzed to identify DEGs between the human non-metastatic cell lines, including normal pancreas cells and primary cells, and liver metastatic PC cell lines (detailed as shown in **Table 1**).

**Figure 1:**
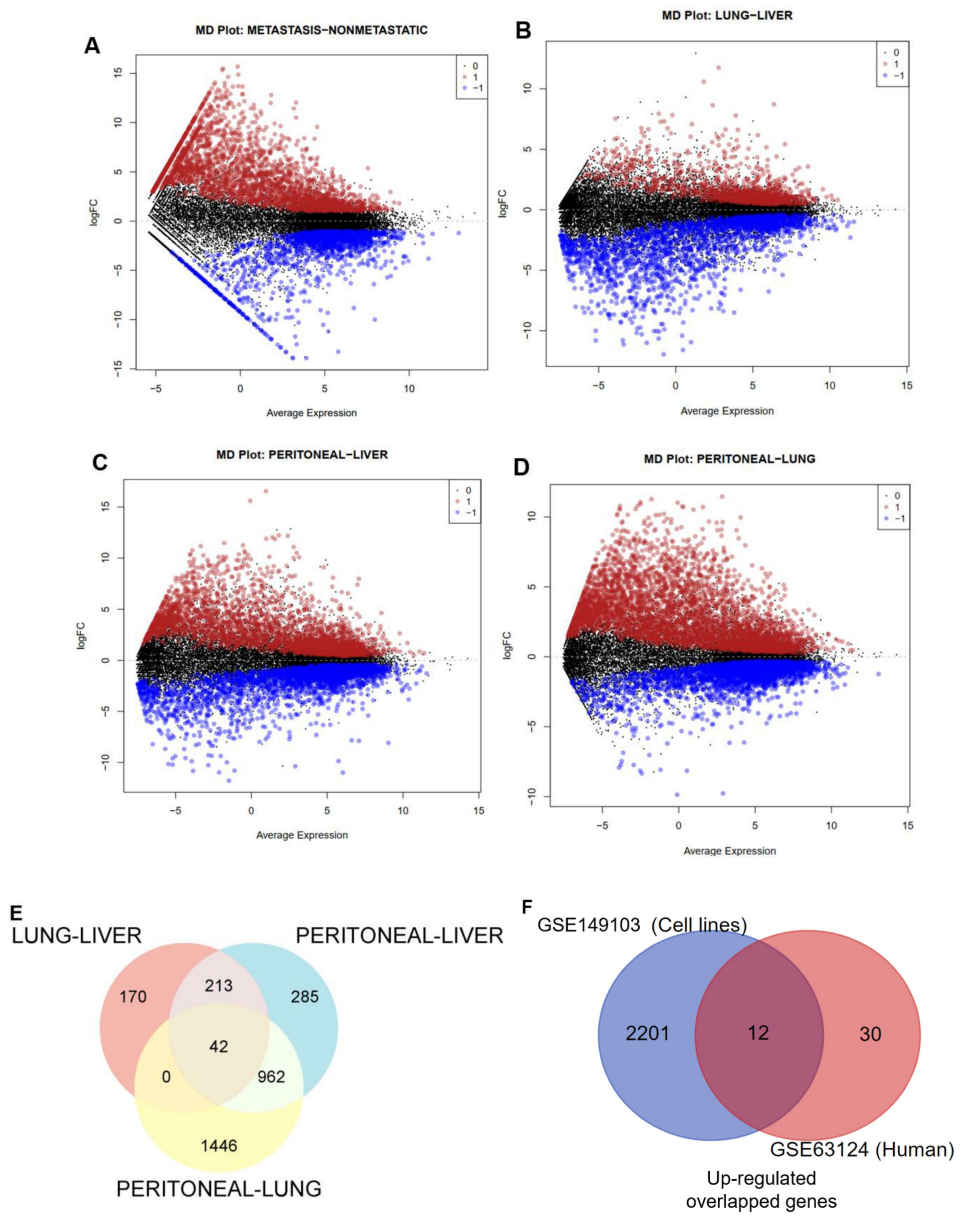
Differentially expressed genes (DEGs) in PC datasets. (**A**) Mean difference plot displaying DEGs between non-metastatic and metastatic PC cell lines. (**B-D**) MD plots presenting DEGs between comparisons of liver (L), lung (Lg), and peritoneal (Peri) metastasis, with upregulated genes (red), downregulated genes (blue), and nonsignificant genes (black). (**E-F**) Venn diagrams depict the overlapped upregulated genes between metastatic sites and cell lines versus human tissues.

The mean difference plot highlighted substantially expressed genes encompassing 2213 upregulated genes and 825 downregulated genes at p-value < 0.01 and |log2 fold change| > 1, as shown in **Fig. 1A**.

Similarly, we analyzed the GSE63124 transcriptomic dataset of PC from human tissue; the information on this dataset is presented in **Table 1**. By using the same cutoff criteria to filter DEGs, **Fig. 1B-D** respectively illustrated 425, 2450, and 1502 upregulated genes in the pair of metastatic sites, namely Lung-Liver, Peritoneal-Lung, and Peritoneal-Liver. We also observed 1043, 943, and 1297 downregulated genes for each pairwise of metastatic sites. Next, we performed Venn diagram analysis to identify the most upregulated genes across these metastatic organs and identified 42 upregulated genes (**Fig. 1E**).

To get more convincing results for determining the most dramatically expressed gene, which was not only presented throughout cell lines but also in virtually all the most common metastatic sites, we then integrated significantly upregulated genes from the cell lines dataset and the overlapped upregulated genes from the human dataset using Venn diagram analysis. As depicted in **Figure 1F**, we finally identified 12 genes.

### ANKRD22 might play a crucial role in metastatic pancreatic cancer

**Figure 2:**
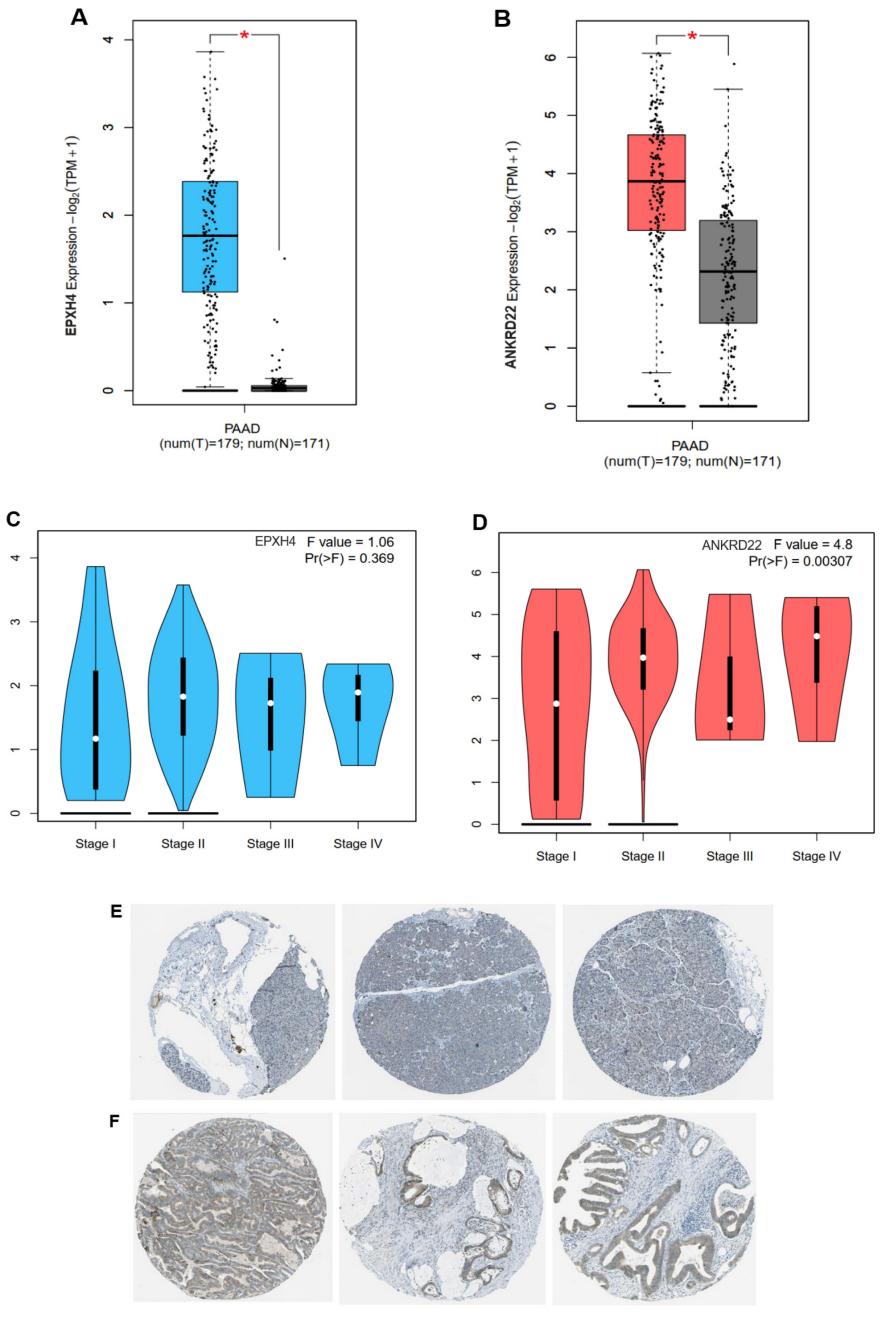
ANKRD22 and EPHX4 were highly expressed in PAAD cohort. (**A-B**) The expression of ANKRD22 or EPHX4 in PAAD group compared to the normal group. (**C-D**) The expressions of ANKRD22 or EPHX4 at various PAAD stages. (**E-F**) ANKRD22 expression in the pancreas and pancreatic cancer tissues at the protein level (HPA database).

**Table 2** shows 5 overlapping upregulated genes, ranked in order based on the Hazard ratio and p-value, including EPHX4, ANKRD22, KIF13B, TMPRSS4, and CCL28, remarkably exacerbated the OS rate of PC patients when their expression was highly exhibited (**Fig. S1**). The seven remaining genes didn't impact the patient outcome (**Table S1**). The two most dramatically different genes were EPHX4 and ANKRD22, with HR = 1.9, p = 0.0023, and HR = 1.7, p = 0.0082, respectively. Consistent with this finding, both genes were highly expressed in the PC group compared to the normal group (**Fig. 2A-B**).

However, when we investigated EPHX4 and ANKRD22 expression in different pathological tumor stages, EPXH4 did not statistically significantly express across cancer stages, F = 1.06, p > 0.05 (**Fig. 2C**), whereas ANKRD22 showed that its presence between tumor stages was significant with F = 4.8 and p < 0.01. Intriguingly, ANKRD22 expressed the highest in stage VI, which has the most dissemination potential, as illustrated in **Fig. 2D**. Additionally, leveraging the Human Protein Atlas database, we delved into the expression of ANKRD22 at the protein level. **Fig. 2E** demonstrated that ANKRD22 protein was presented at a medium level in PC tissues; however, it mainly was not detected in normal pancreas, as shown in **Fig. 2F**.

These results suggest that ANKRD22 might be an oncogene that plays a potential role in PC progression and metastasis. As a result, we chose ANKRD22 as the target gene for subsequent investigations.

### ANKRD22 strongly expressed with mutant KRAS and TP53, leading to a worse OS rate of pancreatic cancer

**Figure 3:**
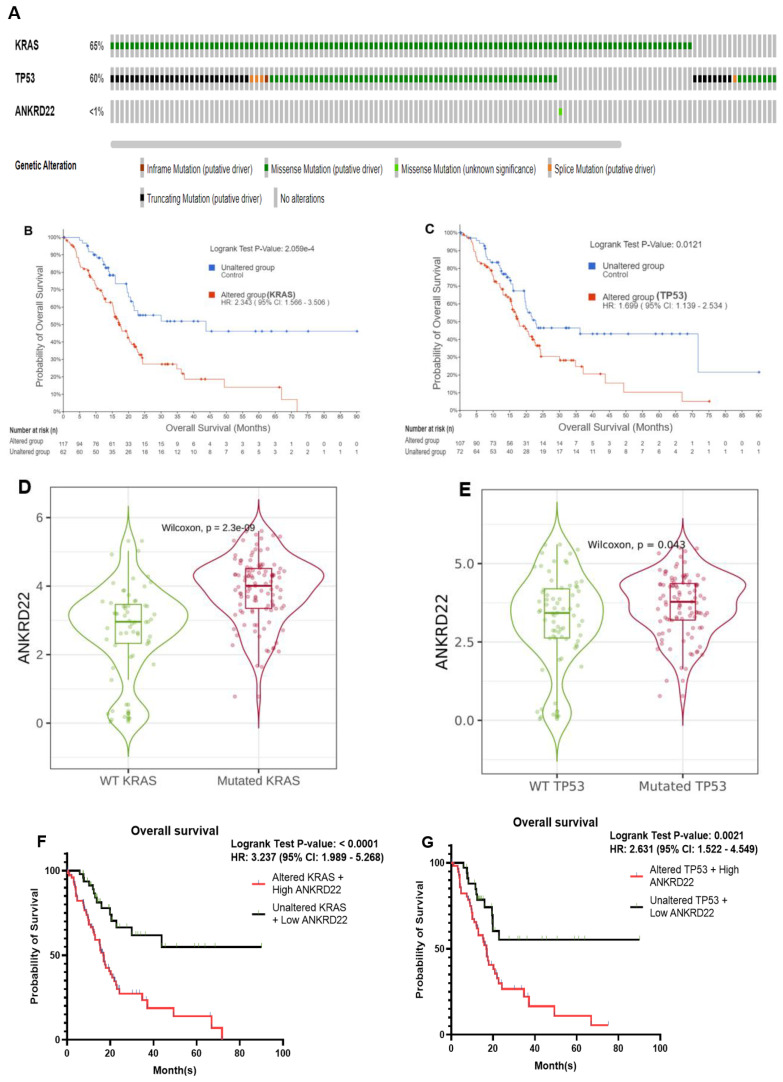
ANKRD22 was highly associated with the most frequently mutated KRAS and TP53 in PAAD, which inversely correlated with OS rate. (**A**) The proportion of genetic alternation of KRAS, TP53, and ANKRD22. (**B-C**) Kaplan-Meier plot showed the OS rate in the mutated KRAS or TP53 group compared to the unaltered group. (**D-E**) ANKRD22 was expressed in the mutated KRAS and TP53 compared to the wild-type (WT) group. (**F-G**) The OS rate of PC patients when high ANKRD22 and mutated KRAS or TP53 were simultaneously expressed compared to low ANKRD22 and variant KRAS or TP53.

The color-coding graph (**Fig. 3A**) visualizes the percentage of KRAS and TP53 mutation in 179 PAAD patients derived from the TCGA database; mutated KRAS and TP53 accounted for 65% and 60%, respectively, and missense mutation was the most frequent alternation in both mutant status, resulting in the notable decrease of survival time than PC patients without mutated KRAS or TP53, HR = 2.34, 95% CI 1.57 - 3.51, p < 0.00021 and HR = 1.7, 95% CI 1.14 - 2.53, p < 0.012, respectively (**Fig. 3B-C**).

On the contrary, **Fig. 3A** shows that ANKRD22 mutation accounted for < 1% of PC cases compared to variant KRAS or TP53 in the same cohort. As revealed in **Fig. 2B** and **Table 2**, ANKRD22 extensively increased in PAAD, leading to a higher risk of PC patients about the survival time, HR = 1.7 and p = 0.0082. Together, this observation implies that the poor OS rate in the presence of ANKRD22 might be well associated with wild-type ANKRD22 rather than mutated ANKRD22. Moreover, we explored ANKRD22 statistically exhibited in variant KRAS and TP53 group compared to the wildtype group (p < 0.05), as shown in **Fig. 3D-E**. This prompted us to question whether mutated KRAS or TP53 co-express ANKRD22 lessens the OS rate. To enclose our hypothesis, we used the same mutated KRAS and TP53 cohorts, then identified which patients highly expressed ANKRD22 and performed Kaplan-Meier curve analysis. As expected, their concurrent expression remarkably worsened this rate compared to unaltered KRAS or TP53 and low ANKRD22 expression, p < 0.001 and HR > 2.6, as illustrated in **Fig. 3F-G**.

Collectively, these observations indicated the possibility that PC patients who harbor mutated KRAS or TP53 might have shorter survival times if they simultaneously expressed high ANKRD22 compared to those who expressed lower ANKRD22, highlighting the crucial role of ANKRD22 in PC.

### ANKRD22 might contribute to the pathways and biological processes of PAAD

Next, we sought the potential biological processes or pathways by which ANKRD22 might contribute to PC initiation.

**Figure 4:**
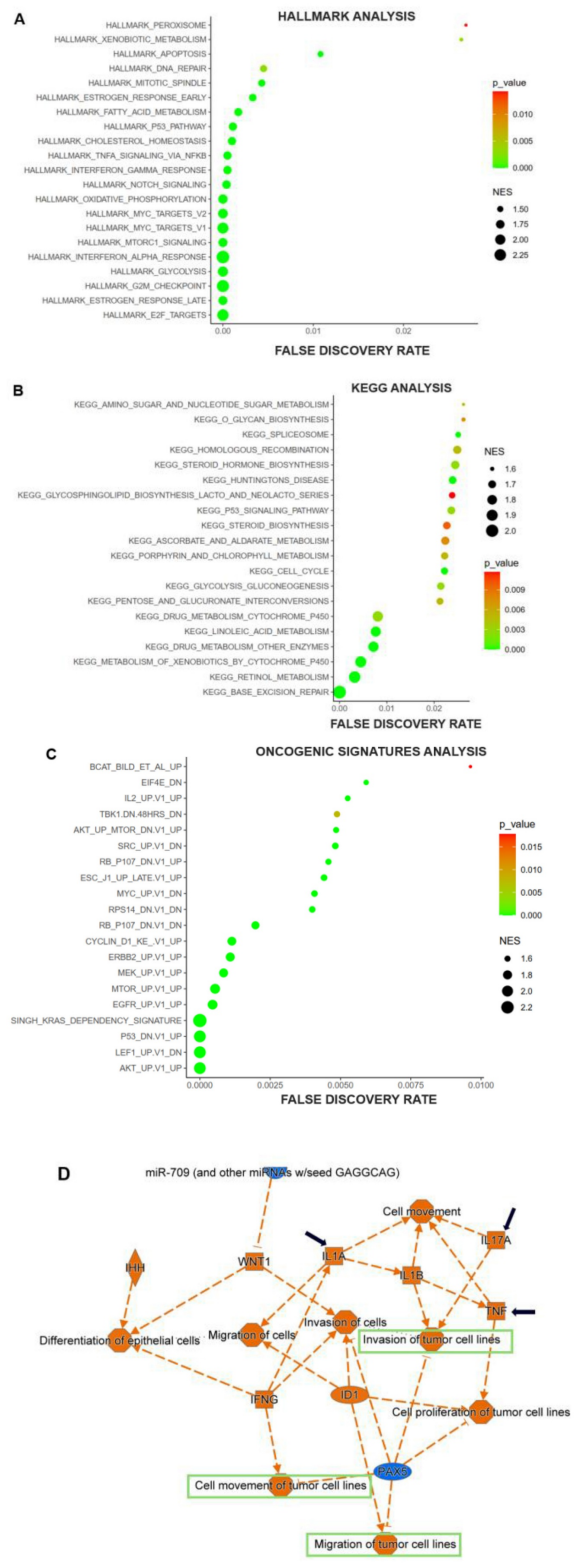
The potential biological processes or pathways ANKRD22 might be involved in in PC. (**A-C**) The significant biological processes or pathways were visualized through Hallmark, KEGG, and Oncogenic signature analysis dot plots. (**D**) The graphical summary represented the major canonical pathways, upstream regulators, diseases, and biological functions using different colors in IPA. Orange represented predicted activation (z-score > 2), while blue represented predicted inhibition (z-score < 2). NES: normalized enrichment score.

Regarding Hallmark analysis, KEGG, and Oncogenic signatures, 21, 30, and 46 datasets met the requirements |NES| > 1, p < 0.05, and FDR < 5%. We chose the first 20 biological states or pathways in each feature to visualize those ANKRD22 might affect. Hallmark results summarized and presented the key biological phenomena or pathways derived from various sources, including KEGG and Oncogenic signatures analyses. As illustrated in **Fig. 4A**, the hallmark *E2F targets* and *G2M checkpoint* were the most significant figures. *TNFα via NF-ĸB, P53 pathway, DNA repair*, and *Apoptosis* were either listed. Besides, KEGG analysis revealed that *Base excision repair* is one of the pathways involved in the DNA damage repair process, and cell_cycle regulation has an impact associated with cell proliferation similar to the effect of E2F targets or the G2M checkpoint mentioned above which might support ANKRD22 in the process of tumorigenesis in PC (**Fig. 4B**). In the Oncogenic signature analysis, we notably observed that the KRAS dependency signature was the most substantial feature driven by high ANKRD22 expression (**Fig. 4C**). This compelling finding reinforces the correlation between ANKRD22 and variant KRAS previously explored **(Fig. 3D)**.

IPA was exploited to provide valuable insights into other processes and pathways under the impact of high ANKRD22 expression. **Fig. 4D** depicts biological functions; we specifically observed some activated functions that contribute to cancer metastasis, such as cell movement, invasion, and migration of tumor cells (denoted by green rectangles). Other factors, including IL17A, IL1, and TNF(denoted by black arrows), were also activated, whose functions might initiate tumor progression.

These described putative pathways and biological processes possibly facilitate ANKRD22 in forming not only PC but also mPC.

### Fostamatinib might be a potential drug targeting ANKRD22

With the results above, ANKRD22 might be a promising theragnostic for mPC. Thus, to examine the drugs that might target ANKRD22, Drugbank and Chembl were used. Although we could not find drugs that directly interact with ANKRD22, we figured out RIPK4 could be ANKRD22's target and interact with fostamatinib, as shown in **Table 3**. This result indicates that if fostamatinib targets RIPK4, it might have some impact on ANKRD22 or its relevant pathways.

**Figure 5:**
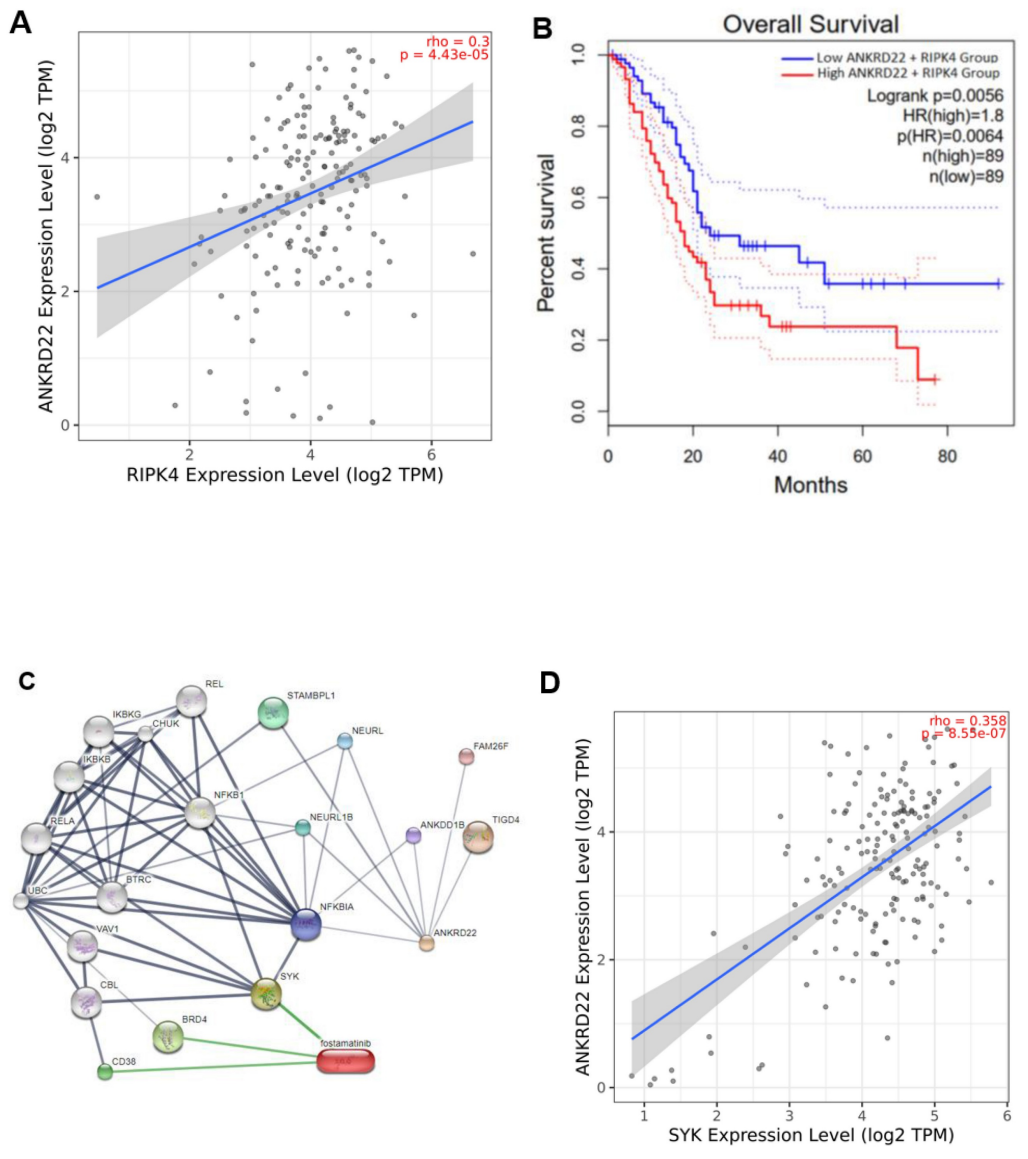
ANKRD22 was positively correlated with SYK and RIPK4 and might be a potential target of fostamatinib. (**A**) The correlation between ANKRD22 and RIPK4. (**B**) The co-expression of ANKRD22 and RIPK4 impacted the OS rate of PAAD. (**C**) The protein-chemical interaction network of ANKRD22 and fostamatinib. (**D**) The correlation between ANKRD22 and SYK.

In order to verify this hypothesis, we investigated the correlation between ANKRD22 and RIPK4. As anticipated, ANKRD22 positively correlated with RIPK4, rho = 0.3 and p < 0.001 (**Fig. 5A**). Furthermore, when ANKRD22 and RIPK4 were concurrently expressed, leading to a poorer OS rate compared to ANKRD22 expression alone (HR = 1.8, p = 0.0056 versus HR = 1.7, p = 0.0082, **Fig. 5B and Table 2**). These findings further supported the ability of fostamatinib to target ANKRD22 and prompted us to utilize STITCH to determine the potential interactions as well as the relevant pathways between ANKRD22 and fostamatinib. **Fig. 5C** shows 21 nodes and 59 edges, p = 0.0014. Of note, the majority of proteins were the components of the nuclear factor kappa B pathway, such as RelA, IKBKG, IKBKB, and NFKBIA. This result might relate to the *TNFα via NF-ĸB* pathway we found in the GSEA analysis. In particular, we observed that SYK (spleen tyrosine kinase), a known target of fostamatinib, was also identified in the network. As a result, we investigated whether SYK and ANKRD22 are associated. Indeed, as shown in **Fig. 5D**, ANKRD22 had a significant favorable association with SYK (rho = 0.36, p < 0.001).

It is reasonable to conclude that ANKRD22 might be a target of fostamatinib, and one of the putative relevant pathways could be NF-ĸB.

Molecular docking emerged as a powerful approach to predict the interactions between proteins (represented as macromolecular targets) and drugs (represented as ligands), especially those interactions for which experiments could not be conducted. Using this method, we performed the binding between fostamatinib and ANKRD22 to understand their interactions and obtain more convincing results to support our proposed hypothesis. Because of the lack of the non-crystal structure of ANKRD22, we utilized Alphafold to get the predicted structure of ANKRD22 and Cavityplus 2022 to generate the proposed binding pockets where fostamatinib might target ANKRD22. Besides, one recent study has examined the AV023 compound's interaction with ANKRD22. Comparing molecular docking results between AV023-ANKRD22 and fostamatinib-ANKRD22 might add more dimensions to the insights of fostamatinib binds to ANKRD22. Thus, we also conducted the molecular docking of AV023 targeted ANKRD22.

Cavityplus yielded four predicted pockets for ANKRD22 and one for AV023. The Druggability and Drug Scores were assessed for each, as shown in **Table 4**. We opted for those whose druggability and drug score were the highest for subsequent analysis. As shown in Table 4, the first binding pocket was chosen (Drug score: -117 and Druggablitiy: medium).

**Figure 6:**
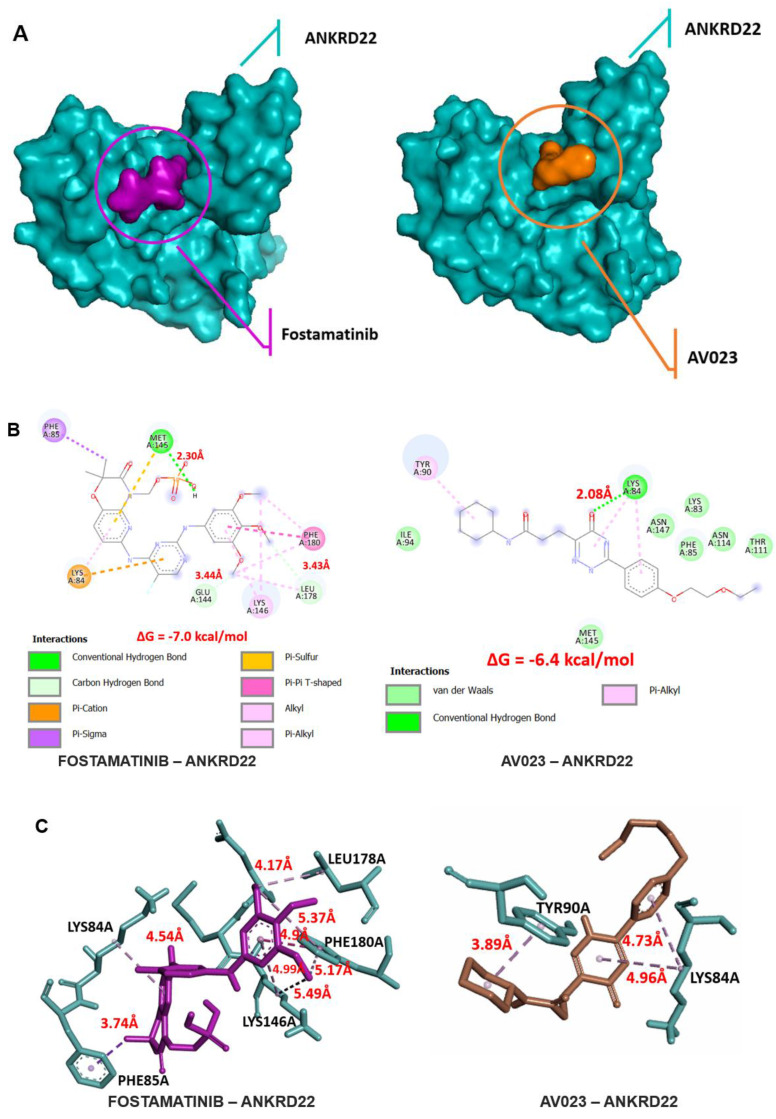
Molecular docking of fostamatinib and AV023 bound to ANKRD22. (**A**) The 3D complexes of fostamatinib and AV023 docked to ANKRD22's binding pocket. (**B**) The 2D diagram of fostamatinib and AV023 in complex with ANKRD22 shows interactions with Hydrogen bonds and binding distances. (**C**) Hydrophobic interactions between fostamatinib and ANKRD22, and AV023 and ANKRD22.

**Fig. 6A** visualizes the poses and potential binding pockets, where fostamatinib or AV023 was bound to ANKRD22 in the 3D conformation. Compared to the AV023-ANKRD22 complex, fostamatinib docked to ANKRD22 with the higher binding free energy value (in absolute terms) ΔG = -7.0 kcal/mol, indicating the stronger affinity binding and formed three hydrogen bonds with MET145 (2.30 Å), GLU144 (3.44 Å) and LEU178 (3.43 Å), AV023 generated ΔG = -6.4 kcal/mol and only one hydrogen bond to LYS84 (2.08 Å) (**Fig. 6B**). Furthermore, the stability of the fostamatinib-ANKRD22 complex was further firmed by eight additional hydrophobic interactions within a distance ranging from 3.74 - 5.49 Å. Conversely, AV023 possessed less hydrophobic interactions at residues LYS84 (4.96 Å), LYS84 (4.73 Å), and TYR90 (3.89 Å) (**Fig. 6C**).

### Fostamatinib more extensively bound to ANKRD22 and yielded better properties compared to those AV023 generated in MD simulation

**Figure 7:**
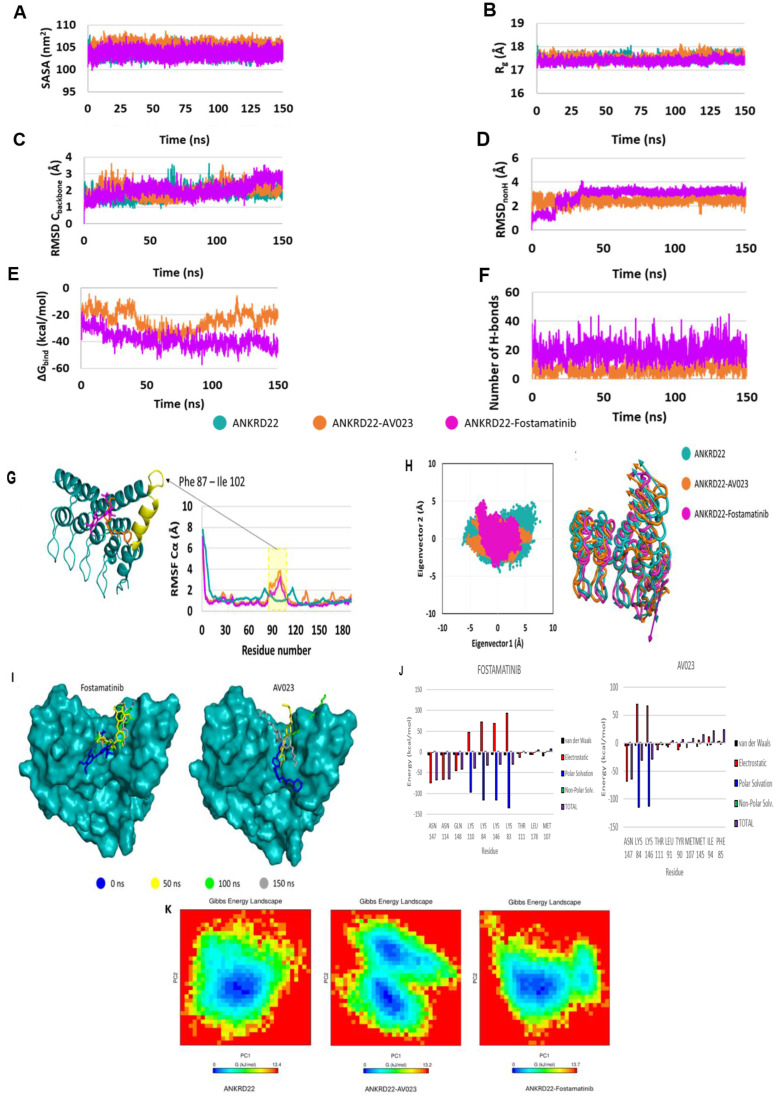
Molecular dynamics simulation of fostamatinib and AV023 to ANKRD22. (**A**) Solvent Accessible Surface Area (SASA), (**B**) Radius of Gyration (Rg), (**C**) Root Mean Square Deviation (RMSD) Cbackbone of protein, (**D**) RMSDnonH of ligand, (**E**) Binding free energy MM/ GBSA, and (**F**) Number of H-bonds of complex analyzed based on 150 ns MD simulation data. (**G**) RMSF Cα values of ANKRD22 residues in MD simulation 150 ns. (**H**) 2D projection of ANKRD22 in complex and apoprotein state calculated against 150 ns MD simulated trajectories, and Porcupine plot of EV1 motion. (**I**) The displacement of ligands in the binding pocket of ANKRD22 was observed at the 150 ns MD simulation. (**J**) Decomposition energy for the top ten amino acid residues corresponding to each complex. Van der Waals: black; Electrostatic: red; Polar Solvation: blue; Non-polar solvation: green and Total: purple). (**K**) The free energy landscapes were obtained at 150 ns MD simulation for free ANKD22 (left panel), ANKD22-AV023 complex (middle panel), and ANKD22-Fostamatinib complex (right panel).

In parallel, we executed molecular dynamics (MD) simulations. This allowed us to delve into fostamatinib's behavior when interacting with ANKRD22 at an atomic level. SASA and R_g_ values presented in **Fig. 7A-B** depict the stability of protein throughout the simulation. The apoprotein (ANKRD22) and the complexes' state (ANKR22-AV023 and ANKRD22-fostamatinib) had similar SASA and R_g_ values throughout 150 ns simulations. As shown in **Fig. 7C**, RMSD C_backbone_ protein values demonstrated that ANKRD22 in complexes were as stable as apoprotein states. RMSD_nonH_ of ligand values showed AV023 was steady during the simulations. In contrast, fostamatinib fluctuated in the first 30 ns and was stable in the last simulations, as illustrated in **Fig. 7D**. Moreover, **Fig. 7E** shows that the fostamatinib-ANKR22 complex yielded higher binding energy and number of H-bonds relative to those in the AV023-ANKRD22 complex, ∆G_bind_ = -38.66 ± 6.09 kcal/mol, 20.65 ± 6.77 H-bonds and ∆G_bind_ = -24.38 ± 7.33 kcal/mol, 8.79 ± 5.36 H-bonds, respectively. **Table 5** shows the top ten H-bonds possessing the highest occupancy rate. Most of the residues were the components of the proposed binding site predicted by Cavityplus. Generally, the fostamatinib-ANKRD22 complex possessed H-bonds whose occupancy rate was greater than the AV023-ANKRD22 complex. TYR90 Side-LIG192 Side, MET107 Side-LIG192 Side, and PHE85 Side-LIG192 Side were the identical pairwise residues between the complexes. The H-bonds possessing the highest occupancy percentage for each complex were PHE85 Side-LIG192 Side (207.01%) and LIG192 Side-THR111 Side (113.03%).

**Fig. 7G** presents the RMSF C_α_ values of residues that fluctuated in MD simulations. The two complexes have fluctuating RMSF values in the α-helix PHE87 - ILE102 compared to the apoprotein state. PCA results (**Fig. 7H, left side**) show that both complexes occupied a minor spatial motion more than the apoprotein state; the ANKRD22-Fostamatinib complex occupied the smallest one. The porcupine plot (**Fig. 7H, right side**) illustrates that the direction and magnitude of movements of the C_backbone_ of complexes and apoprotein states were similar. The residue at α-helix PHE87 - ILE102 fluctuated the most. This observation fitted with the RMSF C_α_ analysis. The displacement of ligands in the binding pocket of ANKRD22 was observed at the 150 ns MD simulations, as indicated in **Fig. 7I**, showing that fostamatinib changed position after 50 ns simulations and then kept this position on during the last simulation period. In contrast, AV023 shifted significantly at the observation times 0, 50, 100, and 150 ns.

Free energy decomposition analyses were performed to determine the key residues in the interaction between ANKRD22 and fostamatinib or AV023. The residues were considered key when the total ∆G_bind_ was less than -1.5 kcal/mol at their position. **Fig. 7J** visualizes the top ten residues for each complex and **Table 6** presents eight key residues, including ASN147, ASN114, GLN148, LYS110, LYS84, LYS146, LYS83 and THR111, in the fostamatinib-ANKRD22 complex, while the AV023-ANKRD22 complex has four residues: ASN 147, LYS84, LYS146, and THR111. Among residues, ASN147 achieved the highest free energy in both complexes.

Furthermore, we leveraged the FEL analysis to precisely portray a protein's most stable conformational ensembles. **Fig. 7K** displays the FELs of the ANKD22, AV023-ANKD22 complex, and fostamatinib-ANKD22 complex, where the deeper blue indicates the most stable conformational state with the lowest energy. Although AV023-ANKRD22 possessed the lowest range of FEL (0 - 13.2 Kj/mol) compared to those of ANKRD22 and fostamatinib-ANKRD22 (0 - 13.4 Kj/mol and 0 - 13.7 Kj/mol, respectively), it had two global minimum and two local minimum illustrated by two clear, distinct basins (**Fig. 7K - middle panel**); this indicates that when AV023 interacts with ANKRD22 might lead to a transition between these two states. Fostamatinib-ANKRD22 had a single global minimum confined within a single basin smaller than the basin in the AV023-ANKRD22 complex. The global minimum of the fostamatinib-ANKRD22 complex also shares a single energy basin with the apo form. FEL analysis indicates that the presence of AV023 alters both the size and position of ANKRD22's energy basin. In contrast, the fostamatinib-ANKRD22 complex remains largely unaffected.

These findings suggest that fostamatinib has a more stable binding to ANKRD22 and holds potential as a promising drug to inhibit PC by suppressing ANKRD22.

## Discussion

Pancreatic cancer is one of the most notorious cancers; its malignancy is reflected by the 5-year survival rate limited to 10% in the US. Besides non-specific markers and symptoms for early detection, insufficient standard treatment also resulted in this dismal rate. Another factor that might contribute to this rate is metastasis. Indeed, the 5-year survival rate dramatically drops to 3% when the primary tumor metastasizes to foreign organs. Gemcitabine is the standard drug for PC therapy so far. However, with its versatile characteristics, PC easily adapts to the Gemcitabine effects, thereby increasing the number of patients harboring Gemcitabine-resistant traits 65, 66. Therefore, more attention must be paid to understanding the fundamental pathways of tumorigenesis in PC, especially for metastasis. Similarly, finding new therapeutic approaches to overcome chemo-resistant status remains an urgent clinical need.

In the present study, we successfully identified ANKRD22, which might be a novel oncogene in mPC. ANKRD22, ankyrin repeat domain 22, is characterized by containing repeat 33-amino acid length ankyrin motifs. This confers ANKRD22 a wide range of interactions with various proteins and might contribute to multiple disease-associated pathophysiological pathways, even cancer, such as lung, colorectal, and ovarian cancer; ANKRD22 functions in different roles, either as a tumor suppressor or supporter. Our study aimed to explore the specific functions of ANKR22 in PC. A comparison analysis was applied for two distinct RNA-seq datasets deriving PC cell lines and PC-driven metastatic tissues. Compared to the normal group, ANKRD22 was extensively expressed in the PC group and highly impacted the OS rate. Our observations implied that ANKRD22 might be the potential marker for PC diagnosis. This is in line with the results of Caba *et al.* After profiling transcriptomic data sequencing from peripheral blood samples of PC patients, the authors identified ANKRD22 as a predictor gene for PC 12. Intriguingly, we found that ANKRD22 expression was statistically significantly different across tumor stages. Its expression in stage IV was the most pronounced, highlighting the prognostic potential of ANKRD22. Mutated genes, including KRAS and TP53, have been reported to be well-involved in PC initiation 67. Our results showed that ANKRD22 was significantly upregulated in the variant KRAS or TP53 group compared to the wildtype group; when ANKRD22 and these oncogenic simultaneously expressed, it substantially worsened the OS rate. These findings further underscored the crucial importance of ANKRD22 in PC. Besides, GSEA analyses indicated that the most significant oncogenic signature of ANKRD22 in PC was KRAS dependency. Our findings were concordant with the observations of Singh *et al.* They revealed that ANKRD22 was highly expressed in KRAS-mutant cell lines 13. The transcription factor E2F1 interferes with cell cycle regulation, resulting in tumor growth. In association with ANKRD22, E2F1 was demonstrated to play an oncogenic role in non-small lung and glioma cancer progression 10, 68. In line with these findings, the *hallmark E2F targets* was the most significant hallmark when ANKRD22 was highly expressed. Furthermore, the *G2M checkpoint* was also identified in this study. These two processes might synergize to get involved in PC promotion. Consequently, ANKRD22 might be a prospective oncogene not only for diagnosis and prognosis but also a candidate for treatment modalities.

With the promising potential of ANKRD22 we had found above, we expected to find a specific drug that can inhibit PC mediated by ANKD22. Although we failed to determine the medicines that interact directly with ANKRD22, we figured out RIPK4, a target of ANKRD22. Our results showed that RIPK4 positively correlated with ANKRD22, and their co-expression shortened the OS rate in PC. Notably, Chembl and Drugbank helped us identify fostamatinib, which targets RIPK4. RIPK4 was a critical molecule possessing high metastatic potential for PC patients characterized by high carcinoembryonic antigen (CEA) and cancer antigen 125-positive (CA125^+^)/CA19-9 levels. Moreover, RIPK4 has been reported to be involved in activating RAF1/MEK/ERK, which has promoted metastasis 69. As shown in IPA analysis, we pinpointed that upon its high expression, ANKRD22 might activate specific metastatic traits encompassing migration and invasion of tumor cells. RIPK4 and ANKRD22 have similar biological functions and a favorable correlation. Therefore, these results suggest that when fostamatinib targets RIPK4, it might interact with ANKRD22. In order to further investigate the interactions between fostamatinib and ANKRD22, the STITCH database was utilized. Interestingly, a well-known target of fostamatinib, SYK- spleen tyrosine kinase, appeared in the ANKRD22-fostamatinib network. Singh *et al.* reported that SYK and ANKRD22 were expressed in Kras-dependent PC cell lines, while their expression was not detected in Kras-independent PC cell lines 13. In our investigation, we found a significant correlation between ANKRD22 and SYK. Hence, ANKRD22 is likely to be targeted by fostamatinib. Fostamatinib is often known by the alternative name R406, which has emerged as a prospective candidate for drug repurposing, especially for cancer treatment. Apart from the original purpose of fostamatinib, inhibiting SYK to treat immune thrombocytopenia disease, there was one current study that has proven that SYK suppression by fostamatinib impacted tumor-associated macrophages behavior and improved the sensitize PC to gemcitabine 70. This interesting result highlighted the importance of fostamatinib in the context of the PC-related immune microenvironment and strengthened our hypothesis that fostamatinib might hinder PC growth. Furthermore, when ANKRD22 was ablated in the Kras-dependent cell lines, one of the key enzymes associated with the apoptosis process, caspase-3, was substantially elevated, as reported in the study of Singh *et al.* 13. Many major constituents of the NF-kB pathway were also presented in STITCH results, which might have an association with *TNFα via NF-ĸB hallmark* listed in GSEA. As well-documented, NF-kB is an anti-apoptosis leading to uncontrolled proliferation of tumor cells, eventually promoting cancer 71, 72. Thus, NF-kB might be a promising pathway that ANKRD22 might exploit for PC tumor initiation. If fostamatinib suppresses ANKRD22, the NF-kB might be impacted the most. It will be crucial to conduct experiments to validate this hypothesis.

Molecular docking and molecular dynamic simulation are valuable methods for exploring the bound modes between ligands and proteins, especially the interaction between drugs and target proteins. Our recent work aimed to demonstrate whether ANKRD22 was a target of fostamatinib owing to these two approaches. While molecular docking helped us predict the binding modes between ANKRD22 and fostamatinib at static conditions, MDs served to validate the resultant molecular docking, particularly at the atomic level, where the free energy will be re-scored and give a more realistic prediction of the interaction. As anticipated, molecular docking and MDs presented the same prediction: fostamatinib bound to ANKRD22 with a high number of hydrogen bonds and a high free energy, ΔG = -7.0 kcal/mol in molecular docking and ∆G_bind_ = -38.66 ± 6.09 kcal/mol in MD simulation. Until recently, only one study has evaluated ANKRD22 docking with another compound, AV023. The comparative analysis showed that fostamatinib-targeted ANKRD22 yielded a stronger affinity than AV023-ANKRD22 binding, which was indicated via the ΔG. As illustrated in the MDs, ANKRD22 contained more H-bonds, and their occupancy rate was higher than that of AV023. In other words, fostamatinib formed with ANKRD22, a more stable complex than AV033 generated. Even though, throughout the 150s of MDs, fostamatinib changed its position in the binding pocket in the first 30s, it kept the last site until the end, whereas AV023 changed continuously. These findings reinforced our hypothesis that fostamatinib might suppress target ANKRD22. As revealed in MD results, the α-helix PHE87 - ILE102 residue fluctuated most during the MD process. This possibly implies that more interactions are likely to occur at this residue. Stated differently, α-helix PHE87 - ILE102 might play a specific function in ANKRD22 protein. In line with the NF-kB pathway explored above, fostamatinib likely impacts ANKRD22 at α-helix PHE87 - ILE102, leading to the activation of the NF-kB pathway, contributing to cell proliferation and apoptosis 73. Besides, for the first time, the key amino acid residues of ANKRD22 were identified and described, including ASN147, ASN114, GLN148, LYS110, LYS84, LYS146, LYS83, and THR111. The presence of these residues enhanced the stabilization of fostamatinib and ANKRD22.

Although our study yielded promising findings, there were some limitations. The correlations between ANKRD22 and RIPK4 or SYK were not extensively strong, just at a moderate level. This result might be affected by the small sample size, range restriction, or outliers. As shown in **Fig. 5A**, the mean expression of ANKRD22 was around 3.5 log_2_TPM; many other values fall far from this point, indicating the outliers. These outliers might cause the mispresent in the correlation. The unavailable crystal structure of ANKRD22 might impact the evaluation of its interaction and fostamatinib. Nevertheless, we used various approaches such as Drugbank, Chembl, STITCH, and Cavityplus 2022, which facilitated us rapidly identifying fostamatinib and further understanding ANKRD22 structure. In particular, the combined use of molecular docking and MD simulations provided a comprehensive understanding of the interactions between our two targets. Despite compelling findings, we utilized bioinformatics approaches. Therefore, we will conduct corresponding experiments to validate these results further. Owing to the results, such as ANKRD22 remarkably expressed in the tumor group, potentially contributing to the NF-kB pathway and metastatic process, the qPCR and western blot could be conducted to investigate relevant components. Fostamatinib would be used to validate the potency *in vitro* and *in vivo*. Hopefully, this study will not only provide the fundamental concepts of ANKRD22's role in PC and even metastatic PC but also place the first brick for the invention of ANKRD22-targeted anti-cancer therapy.

## Supplementary Material

Supplementary figures and table.

## Figures and Tables

**Table 1:** Details of pancreas cells, PC cell lines, and samples of metastatic sites

GEO	Platform	Cell lines/Patient ID	Property/Metastatic sites	Replicates/Number of samples	Group
GSE149103	GPL20795	HPNE	Human pancreas normal epithelial cells	2	NONMETASTATIC
		PANC-1	Primary pancreatic cancer cells	2	
		CAPAN-1	Metastatic pancreatic cancer cells	2	METASTASIS
GSE63124	GPL11154	A38	Liver	2	LIVER
			Lung	2	LUNG
			Peritoneal	2	PERITONEAL
		A13	Lung	2	LUNG

**Table 2:** The overlapping upregulated genes with the hazard ratio and p-value in the PAAD cohort

SYMBOL	Gene name	*p-value*	HR
*EPHX4*	Epoxide hydrolase 4	0.0023	1.9
*ANKRD22*	Ankyrin repeat domain 22	0.0082	1.7
*KIF13B*	Kinesin family member 13B	0.013	1.7
*TMPRSS4*	Transmembrane serine protease 4	0.015	1.7
*CCL28*	C-C motif chemokine ligand 28	0.047	1.5

**Table 3:** ANKRD22 targets and related drugs retrieved from the DrugBank and Chembl databases

Database	ANKRD22 targets	UNIPROT ID	Drug interactions with/Quantity of drugs or compounds	DRUG_ID	Actions/Assay description
Drugbank	Receptor-interacting serine/threonine-protein kinase 4 (RIPK4)	P57078	Fostamatinib/01	Drugbank_ID: DB12010	Inhibitor
Chembl	Receptor-interacting serine/threonine-protein kinase 4 (RIPK4)	P57078	Fostamatinib/245	Chembl_ID: CHEMBL475251	Binding constant for RIPK4 kinase domain

**Table 4:** Characteristics of predicted binding pockets of ANKRD22 with the fostamatinib and AV023 ligands

	Predicted binding pocket	Pred max pKd	Pred avg pKd	Drug Score	Druggability
FOSTAMATINIB	1	9.67	5.93	-117.00	Medium
	2	7.48	5.18	-1083.00	Weak
	3	5.83	4.62	-1348.00	Weak
	4	5.59	4.54	-1458.00	Weak
AV023	1	11.15	6.44	402.00	Medium

* Pred max pKd: predicted maximum dissociation constant; Pred avg pKd: predicted average dissociation constant.

**Table 5:** The occupancy rates of the top ten H-bonds occupying the highest occupancy rate in MD simulations of fostamatinib and AV023 to ANKRD22

Ligands	Donor	Acceptor	Occupancy rates (%)
FOSTAMATINIB	PHE85-Side	LIG192-Side	207.01%
	LIG192-Side	PHE85-Side	165.33%
	GLN148-Side	LIG192-Side	132.02%
	SER149-Side	LIG192-Side	125.84%
	TYR90-Side	LIG192-Side	114.24%
	LIG192-Side	GLY103-Main	96.45%
	ILE93-Side	LIG192-Side	92.14%
	MET107-Side	LIG192-Side	77.95%
	LYS146-Side	LIG192-Side	77.58%
	GLN148-Main	LIG192-Side	73.47%
AV023	LIG192-Side	THR111-Side	113.03%
	LYS84-Side	LIG192-Side	99.27%
	TYR90-Side	LIG192-Side	79.24%
	MET107-Side	LIG192-Side	40.30%
	PHE85-Side	LIG192-Side	38.65%
	LIG192-Side	LYS83-Main	36.85%
	LIG192-Side	TYR90-Side	35.06%
	THR111-Side	LIG192-Side	32.65%
	TYR104-Side	LIG192-Side	32.63%
	LIG192-Side	TYR104-Side	31.50%

**Table 6:** Free energy decomposition for key residues in MD simulations of fostamatinib and AV023 to ANKRD22

Ligands	Residue	van der Waals	Electrostatic	Polar Solvation	Non-Polar Solv.	TOTAL
FOSTAMATINIB	ASN 147	-7.18	-74.36	-0.15	0.00	-67.17
	ASN 114	-6.82	-66.43	-5.03	0.08	-64.79
	GLN 148	-6.32	-44.29	-7.18	0.32	-41.67
	LYS 110	-6.11	46.51	-97.04	0.37	-38.46
	LYS84	-5.76	72.16	-115.59	0.59	-31.77
	LYS 146	-6.00	68.71	-116.03	0.69	-29.65
	LYS83	-7.15	92.62	-133.91	0.41	-29.58
	THR 111	-3.60	-12.72	-1.20	0.12	-2.12
AV023	ASN 147	-6.06	-68.41	-3.92	0.08	-63.92
	LYS84	-4.77	69.80	-114.24	0.74	-30.78
	LYS 146	-5.55	66.34	-112.78	0.82	-28.78
	THR 111	-3.72	-11.97	-2.07	0.18	-2.29

## Abbreviations

2D: two-dimensional
3D: three-dimensional
AKT: serine/threonine kinase
ANKRD22: ankyrin repeat domain 22
CPM: count per million
DEGs: differentially expressed genes
EMT: epithelial-mesenchymal transition
EPHX4: epoxide hydrolase 4
ERK: extracellular signal-regulated kinase
FC: fold change
FDA: Food and Drug Administration
FDR: false discovery rate
FEL: free energy landscape
GEO: gene expression omnibus
GEPIA: gene expression profiling interactive analysis
GSEA: gene set enrichment analysis
GTEx: genotype-tissue expression projects
HCC: hepatocellular carcinoma
HPA: the human protein Atlas
HRs: hazard ratios
IHC: immunohistochemical
IPA: ingenuity pathway analysis
JAK: janus kinase
MAPK: mitogen-activated protein kinase
MDs: molecular dynamic simulation
mPC: metastatic pancreatic cancer
NES: normalized enrichment score
OS: overall survival
PAAD: pancreatic ductal adenocarcinoma
PC: pancreatic cancer
PI3K: phosphatidylinositol 3-kinase
RIPK4: serine/threonine-protein kinase 4
RNA-Seq: RNA-sequence
STAT: signal transduction and activator of transcription
STITCH: search tool for interacting chemicals
SYK: spleen tyrosine kinase
TCGA: the cancer genome atlas
