# Supplementary material for: In Silico Identification of ANKRD22 as a Theragnostic Target for Pancreatic Cancer and Fostamatinib's Therapeutic Potential
Source: Int J Med Sci. 2025 Mar 19;22(8):1885–904. doi: 10.7150/ijms.105193 (PMC11983316; doi:10.7150/ijms.105193)
Supplement: Supplementary file 1 — Supplementary figures and table. [file ijmsv22p1885s1.pdf]

SUPPLEMENTARY FIGURE

In Silico Identification of ANKRD22 as a Theragnostic Target for Pancreatic Cancer and Fostamatinib's Therapeutic Potential

Authors:

- **Huong Thi Luu Kim Huynh[1]**
- **Hendrick Gao-Min Lim[2,3]**
- **Yuan-Chii Gladys Lee[2]**
- **Thien-Vy Phan[4]**
- **Thanh-Hoa Vo[5]**
- **Chien-Hsin Chen[6,7]**
- **Alexander T.H. Wu[1,2,8,9,10,11]**

Affiliations

1. International PhD Program for Translational Science, College of Medical Science and Technology, Taipei Medical University, Taipei 11031, Taiwan
2. Graduate Institute of Biomedical Informatics, College of Medical Science and Technology, Taipei Medical University, Taipei 11031, Taiwan
3. Department of Medical Research, Tzu Chi Hospital Indonesia, Pantai Indah Kapuk, Greater Jakarta, Indonesia 14470
4. Department of Pharmacy, Nguyen Tat Thanh University, Ho Chi Minh City 700000, Vietnam
5. University of Health Sciences, Vietnam National University Ho Chi Minh City, Ho Chi Minh City 700000, Vietnam
6. Department of Surgery, College of Medicine, Taipei Medical University, Taipei 11031, Taiwan
7. Department of Colorectal Surgery, Wan Fang Hospital, Taipei Medical University, Taipei 11031, Taiwan
8. TMU Research Center of Cancer Translational Medicine, Taipei Medical University, Taipei 11031, Taiwan chaw1211@tmu.edu.tw
9. The PhD Program of Translational Medicine, College of Medical Science and Technology, Taipei Medical University, Taipei 11031, Taiwan
10. Graduate Institute of Medical Sciences, National Defense Medical Center, Taipei 114, Taiwan
11. Taipei Heart Institute (THI), Taipei Medical University. Taipei, Taiwan

Corresponding authors: Alexander T.H. Wu and Chien-Hsin Chen

Supplementary table S1.

The overlapping upregulated genes with the hazard ratio and p-value in the PAAD cohort (non-significant results)

SYMBOL	Gene name	*p-value*	HR
SOWAHB	Sosondowah ankyrin repeat domain family member B	0.18	1.3
SPDEF	SAM pointed to the domain containing the ETS transcription factor	0.22	1.3
EPCAM	Epithelial cell adhesion molecule	0.26	1.3
SLC28A3	Solute carrier family 28-member 3	0.37	1.2
FEZF1	FEZ family zinc finger 1	0.37	0.83
ACE2	Angiotensin-converting enzyme 2	0.55	1.1
TMPRSS2	Transmembrane serine protease 2	0.72	1.1

Supplementary Figure 1.

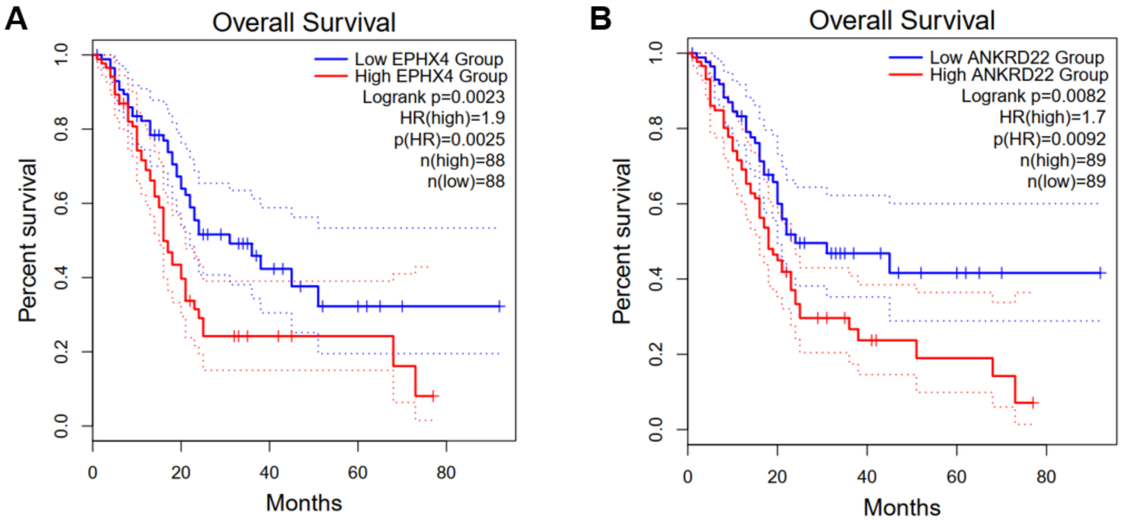

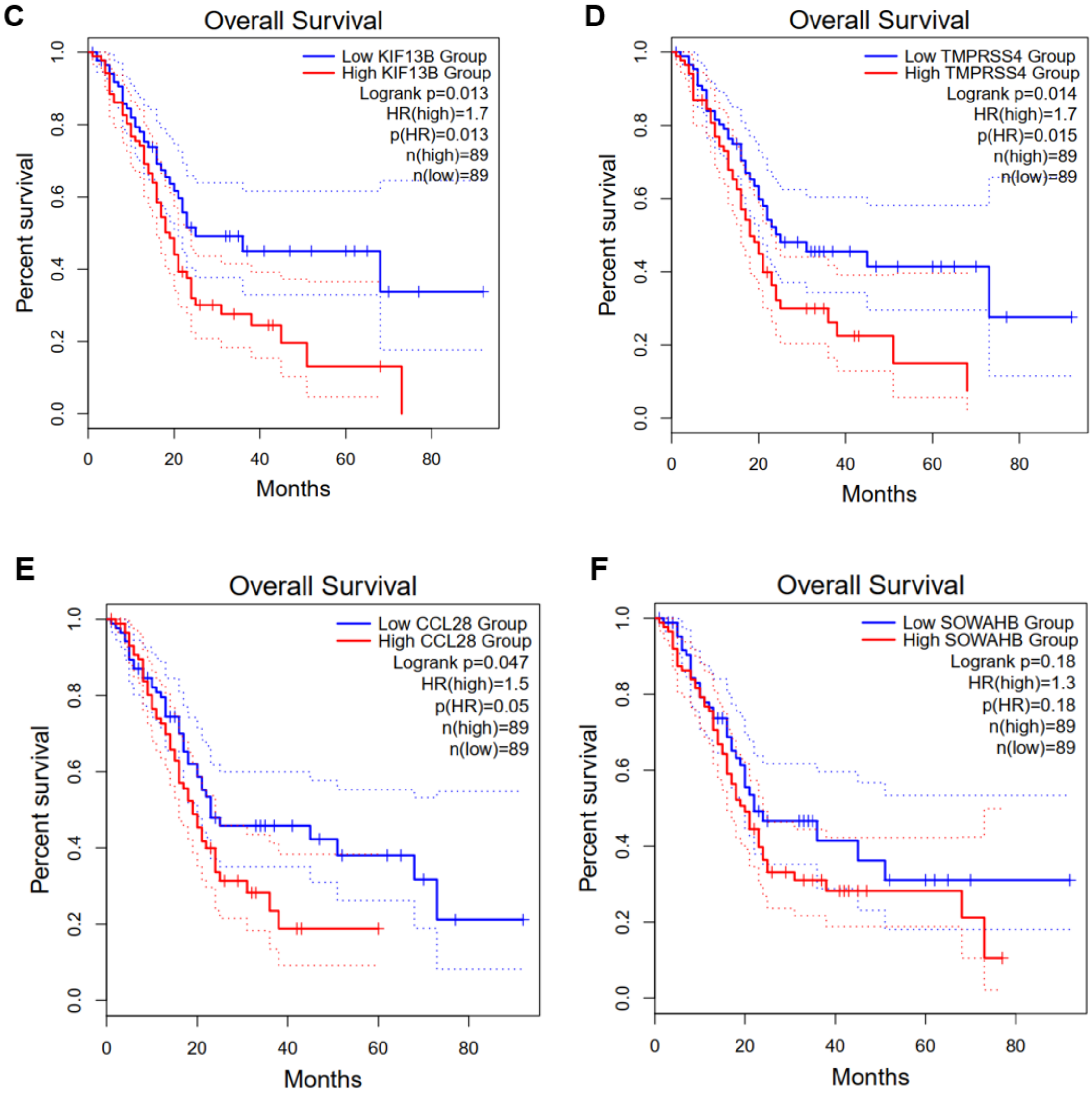
C
Overall Survival
Low KIF13B Group
High KIF13B Group
Logrank p=0.013
HR(high)=1.7
p(HR)=0.013
n(high)=89
n(low)=89
Percent survival
Months
D
Overall Survival
Low TMPRSS4 Group
High TMPRSS4 Group
Logrank p=0.014
HR(high)=1.7
p(HR)=0.015
n(high)=89
n(low)=89
Percent survival
Months
E
Overall Survival
Low CCL28 Group
High CCL28 Group
Logrank p=0.047
HR(high)=1.5
p(HR)=0.05
n(high)=89
n(low)=89
Percent survival
Months
F
Overall Survival
Low SOWAHB Group
High SOWAHB Group
Logrank p=0.18
HR(high)=1.3
p(HR)=0.18
n(high)=89
n(low)=89
Percent survival
Months

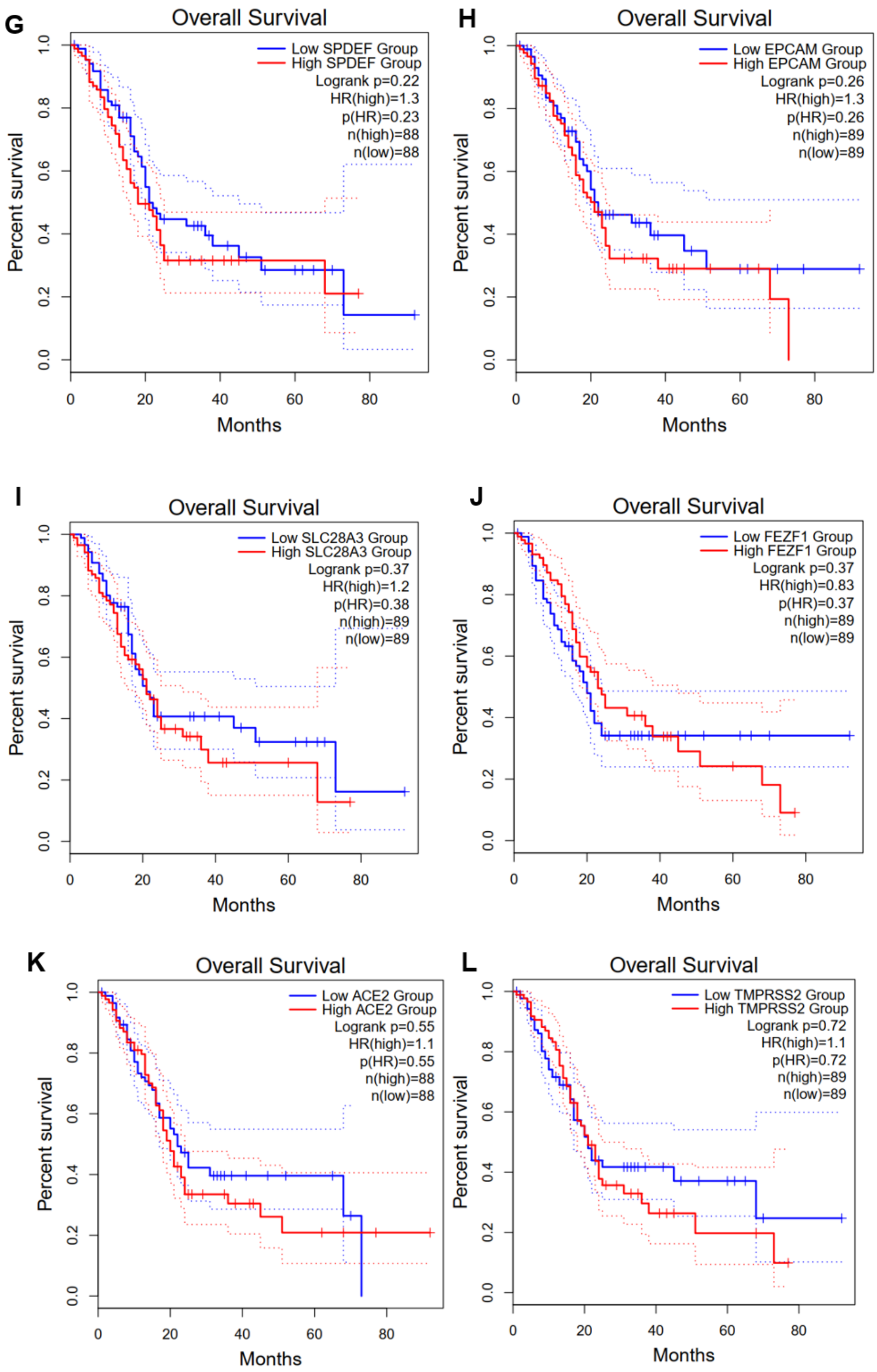
G
Overall Survival
Low SPDEF Group
High SPDEF Group
Logrank p=0.22
HR(high)=1.3
p(HR)=0.23
n(high)=88
n(low)=88
Percent survival
Months
H
Overall Survival
Low EPCAM Group
High EPCAM Group
Logrank p=0.26
HR(high)=1.3
p(HR)=0.26
n(high)=89
n(low)=89
Percent survival
Months
I
Overall Survival
Low SLC28A3 Group
High SLC28A3 Group
Logrank p=0.37
HR(high)=1.2
p(HR)=0.38
n(high)=89
n(low)=89
Percent survival
Months
J
Overall Survival
Low FEZF1 Group
High FEZF1 Group
Logrank p=0.37
HR(high)=0.83
p(HR)=0.37
n(high)=89
n(low)=89
Percent survival
Months
K
Overall Survival
Low ACE2 Group
High ACE2 Group
Logrank p=0.55
HR(high)=1.1
p(HR)=0.55
n(high)=88
n(low)=88
Percent survival
Months
L
Overall Survival
Low TMPRSS2 Group
High TMPRSS2 Group
Logrank p=0.72
HR(high)=1.1
p(HR)=0.72
n(high)=89
n(low)=89
Percent survival
Months

Figure S1. The overall survival rate of PC patients is based on overlapping upregulated gene expression. (A-L). OS rate for EPHX4, ANKRD22, KIF13B, TMPRSS4, CCL28, SOWAHB, SPDEF, EPCAM, SLC28A3, FEZF1, ACE2, and TMPRSS2, respectively.
